# Triple Jeopardy? Stress Among Dementia Caregivers Through the Lens of Intersectionality

**DOI:** 10.1093/geroni/igaa057.196

**Published:** 2020-12-16

**Authors:** Ruotong Liu, Iris Chi

**Affiliations:** University of Southern California, Los Angeles, California, United States

## Abstract

Despite the benefits to economy and public health, caregivers are negatively affected by caregiving activities. Dementia caregivers, compared to other caregivers, are experiencing higher levels of stress, due to reasons such as the care recipients’ changes in personalities and behaviors. Previous studies have documented differences in caregiver stress across gender and racial/ethnic groups. However, few studies have looked into caregiving differences within both gender and race/ethnicity through an intersectionality framework. This paper seeks to explore what are the differences in caregiving stress across the intersectionality of race and gender. Using Round 5 and Round 7 of NSOC and NHATS data, we examined differences in caregiver stress across and within different gender and racial/ethnic groups in terms of financial, emotional, and physical stress. 1,206 caregivers were included with 61% female, 50% White, and 32% Black caregivers. Logistic regression results indicate that female is less likely to have financial stress, but more likely to experience emotional stress. Compared to White, Black caregivers are worse off financially but better off physically. Both Black and other racial/ethnic caregivers are less likely to have emotional stress. Within the intersectionality framework, compared to White female, Black male are 3.4 times more likely to experience financial stress, all the other groups are 38%-71% less likely to have emotional stress, and Black female are 53% less likely to have physical stress compared to White female. The findings highlighted that in order to develop more effective interventions or policies, unique areas are to be focused for different population subgroups.

